# Anatomic Variations of Renal Arteries as an Important Factor in the Effectiveness of Renal Denervation in Resistant Hypertension

**DOI:** 10.3390/jcdd10090371

**Published:** 2023-08-29

**Authors:** Karol Kasprzycki, Paweł Petkow-Dimitrow, Agata Krawczyk-Ożóg, Stanisław Bartuś, Renata Rajtar-Salwa

**Affiliations:** 1 Department of Cardiology and Cardiovascular Interventions, University Hospital, 30-688 Krakow, Poland; 2 2nd Department of Cardiology, Jagiellonian University Medical College, 30-688 Krakow, Poland; 3 Department of Anatomy, Jagiellonian University Medical College, 33-332 Krakow, Poland

**Keywords:** resistant hypertension, renal denervation, renal arteries anatomy

## Abstract

Hypertension remains the leading cause of death worldwide. Despite advances in drug-based treatment, many patients do not achieve target blood pressure. In recent years, there has been an increased interest in invasive hypertension treatment methods. Long-term effects and factors affecting renal denervation effectiveness are still under investigation. Some investigators found that the renal arteries’ morphology is crucial in renal denervation effectiveness. Accessory renal arteries occur in 20–30% of the population and even more frequently in patients with resistant hypertension. Diversity in renal vascularization and innervation may complicate the renal denervation procedure and increase the number of people who will not benefit from treatment. Based on previous studies, it has been shown that the presence of accessory renal arteries, and in particular, the lack of their complete denervation, reduces the procedure’s effectiveness. The following review presents the anatomical assessment of the renal arteries, emphasizing the importance of imaging tests. Examples of imaging and denervation methods to optimize the procedure are presented. The development of new-generation catheters and the advancement in knowledge of renal arteries anatomy may improve the effectiveness of treatment and reduce the number of patients who do not respond to treatment.

## 1. Introduction

**Figure 1:**
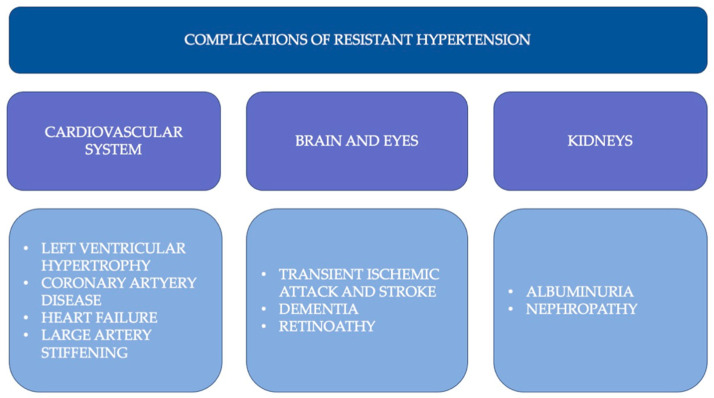
Complications of resistant hypertension in different organs [1,2].

**Figure 2:**
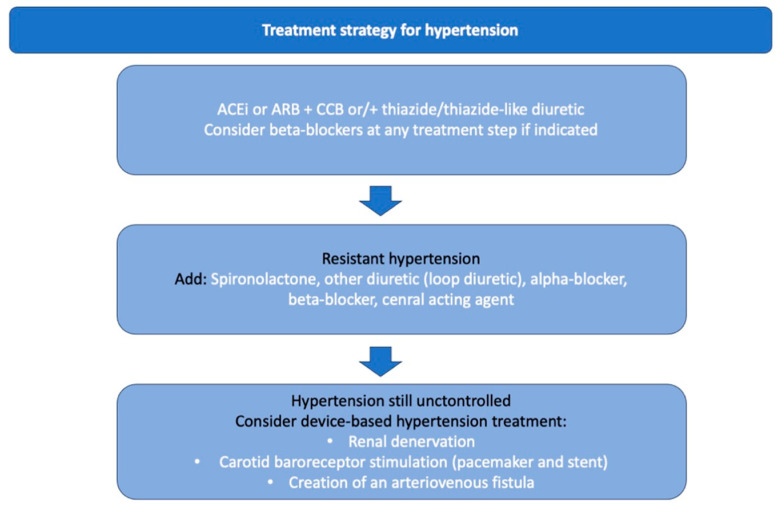
Treatment strategy for hypertension [1,5].

Hypertension is one of the most prominent modifiable risk factors for cardiovascular diseases (CVDs). According to the World Health Organization (WHO), hypertension also remains the most common cause of premature death worldwide. The relationship between high blood pressure (BP) and mortality and cardiovascular events, e.g., myocardial infarction, stroke, and peripheral arterial disease, is linear across all age groups for both men and women (Figure 1). A systolic blood pressure decrease of 10 mmHg reduces cardiovascular events by 25–30% [1,2]. Despite significant advancements in drug-based therapy, many patients do not reach target blood pressure levels, and gradual escalation therapy is necessary (Figure 2). Furthermore, the use of drugs is often associated with side effects and may impair compliance. As a result, new therapy methods are still being searched for, which would have a lesser impact on the patient’s lifestyle. Resistant hypertension (RTH) is defined as insufficient blood pressure control and maintaining blood pressure above 140/90 mmHg when using three drugs, including diuretics, correctly combined and in full doses, which is confirmed with 24 h Ambulatory Blood Pressure Monitoring (ABPM) or home blood pressure measurements. It is also necessary to exclude pseudo-resistant hypertension, e.g., poor adherence to medication, a white phenomenon lab coat, poor measurement technique, calcification of the brachial artery, and secondary causes of hypertension, including obstructive sleep apnea, kidney disease, renal artery stenosis, and primary hyperaldosteronism. Treatment of hypertension includes three areas: lifestyle changes, drug therapy, and interventional treatment, which is especially useful in patients with resistant hypertension [1]. The most widely used invasive treatment method for hypertension is percutaneous renal denervation (RDN). According to the recent guidelines of the European Society of Cardiology and the European Society of Hypertension (ESC/ESH), percutaneous denervation of the renal arteries is not recommended for the routine management of hypertension. However, it is allowed to be used in specialized centers by experienced interventional cardiologists in patients in whom multidrug pharmacotherapy of hypertension is ineffective [1,3]. The results of the Simplicity 3 study significantly influenced the current recommendations. The Simplicity 3 clinical trial failed to reveal a significant decrease in blood pressure twelve months after the RDN procedure [4]. However, several large, well-designed, randomized clinical trials have been conducted recently, showing a significant reduction in BP in patients undergoing RDN [5,6]. To improve the effectiveness of the renal denervation procedure, reasons that may reduce the response to the treatment are sought. Lack of ablation of the accessory renal arteries (ARA) has been postulated as one of the possible causes of non-response to treatment. This paper presents the current state of knowledge on the impact of accessory renal arteries on the effectiveness of renal artery denervation [7,8,9].

## 2. Anatomy and Pathophysiology

Renal arteries are paired arteries originating from the lateral part of the abdominal aorta, typically at the L1/L2 intervertebral disk level, inferior to the take-off of the superior mesenteric artery. Renal arteries are, on average, 4 to 6 cm long and 5 mm to 6 mm in diameter [10]. They are the only vascular supply to the kidneys. 

Kidneys are richly innervated organs. They receive afferent and efferent nerves, of which the efferent is strictly sympathetic. They comprise the renal plexus and receive inputs from the celiac, aortic, renal plexuses, and the least splanchnic nerves [11,12]. Many studies have shown that excessive sympathetic nervous system (SNS) activity may increase blood pressure. The critical function of the SNS in hypertension pathophysiology has been analyzed by measuring its activity in hypertensive individuals and examining blood pressure changes after manipulating sympathetic activity. Several studies have found more elevated levels of catecholamines in subjects with hypertension. Moreover, a direct correlation between blood pressure and sympathetic activity has been shown in patients with severe hypertension [13,14,15].

The renal sympathetic nervous system impacts blood pressure through two pathways. It supplies the kidneys via a network of efferent, noradrenergic, sympathetic fibers that are located in the adventitia of the renal arteries. It also returns signals to the central nervous system through the afferent sympathetic nerve fibers located in the adventitia. Stimulation of the renal efferent sympathetic nerves increases the tubular reabsorption of water and sodium ions and vasoconstriction of the renal vessels. A decrease in renal blood flow and glomerular filtration leads to an increase in the secretion of renin and norepinephrine, causing an increase in blood pressure. However, based on past research, the afferent nerves play a crucial role in the blood pressure effects of renal denervation. Stimulation of renal afferent sympathetic nerves as a result of renal ischemia directly affects the activity of the posterior hypothalamus, leading to increased secretion of norepinephrine and angiotensin II [13,16].

## 3. The Course of the Procedure

Percutaneous renal sympathetic denervation (RDN) is a minimally invasive method to treat resistant hypertension. The procedure aims to damage the sympathetic, efferent, and afferent fibers that run in the renal arteries adventitia, which is supposed to decrease sympathetic stimulation and, consequently, blood pressure.

RDN is usually performed using the femoral artery approach in local anesthesia. The catheter is inserted into the lumen of the renal artery, where, using specified intervention, the nerve fibers located in the adventitia are damaged. 

In clinical practice, three methods of denervation are used:

- radiofrequency (RF) ablation uses multi-electrode catheters or intravascular balloons; during the intervention, radiofrequency pulses are applied, inducing the burning of the nerve fibers in the renal arteries adventitia,
- ultrasound ablation, which uses acoustic wave energy delivered intra- or extravascularly, and
- pharmacological ablation, which uses microinjections of the drug into the vessel wall and perivascular space [3,13,16,17,18,19,20,21,22].

## 4. Accessory Renal Arteries

**Figure 3:**
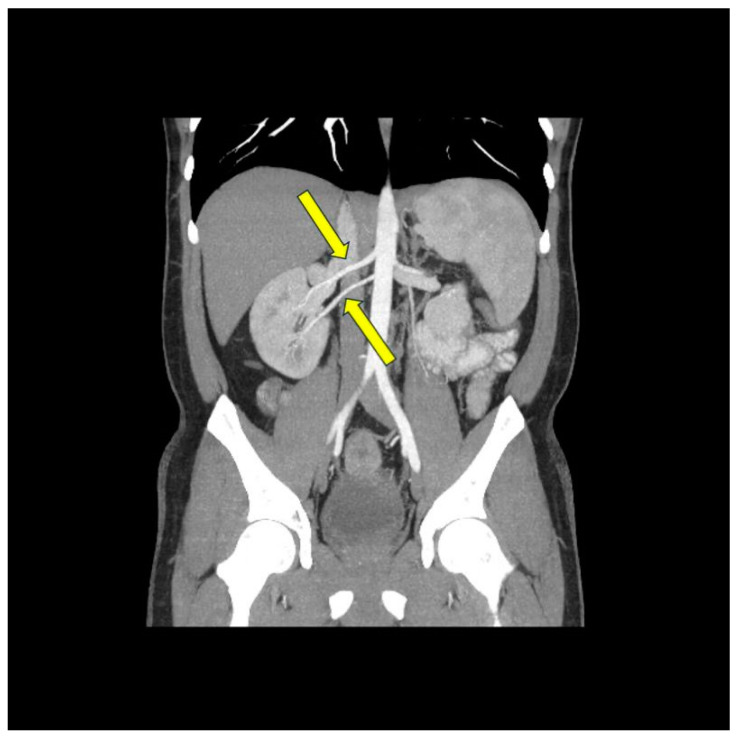
Contrast-enhanced computed tomography scan showing multiple right renal arteries (yellow arrows).

Based on previous experience gained during clinical trials and procedures performed in many centers worldwide, many patients after the RDN procedure do not show a decrease in blood pressure. Studies showed that 10% to 13% of the patients did not respond to therapy [23,24,25]. That prompts researchers to look for factors responsible for the lack of response to the procedure and to create appropriate criteria for selecting patients who will benefit from renal denervation. Many authors state that the nonresponse to the RDN is connected with the renal arteries’ anatomy [7,8,9]. Most randomized controlled trials initially enrolled patients with specified renal anatomy [4,26,27]. The exclusion criteria were the prevalence of accessory renal arteries (Figure 3), a too-small diameter of the renal artery (≤4 mm), a too-short length of the main renal artery (<20 mm), and renal atherosclerotic disease, including previous angioplasty or stenting. It is worth noting that according to autopsy data, the prevalence of accessory renal arteries is high in the population and is about 20–30%, which resulted in the exclusion of a large number of potential patients [4,26,27,28,29,30,31]. 

Additionally, accessory renal arteries are present even more often in patients with resistant hypertension than in the general population. It has been suggested that accessory renal arteries play a role in hypertension pathophysiology via activation of the renin–angiotensin–aldosterone system [7,29,30,32]. Rimoldi et al. retrospectively studied 941 consecutive hypertensive individuals who underwent coronary angiography and concomitant selective angiography of renal arteries. They found that only 52% were anatomically suitable for sympathetic RDN according to the criteria used in the SYMPLICITY HTN 3 study [4,26]. The survey by Rimoldi et al. showed that the number of patients who meet the anatomical criteria for renal artery denervation is significantly lower than previously thought. Previous studies have estimated that 63–90% of patients may be qualified for the procedure [24,26,30,31,32,33,34].

### 4.1. Prevalence and Methods of Imaging

Accessory renal arteries are a common anatomical variant of renal vascularity. ARAs are defined as vessels other than the main renal artery originating from the abdominal aorta, which vascularizes the kidney either by passing through its hilum or directly entering its pole.

The prevalence of accessory renal arteries, according to the available literature, ranges between 11% and 61% and varies significantly between different authors [26,27,28,29,30,34,35]. During the RDN procedure, the early branches of the main renal artery, defined as arteries arising from the main renal arteries within 20 mm of the ostium, are as crucial as the accessory renal arteries. Studies conducted so far, mainly using imaging studies concerned with patients with resistant hypertension, suggest that accessory renal arteries may significantly impact the occurrence and possibilities of invasive treatment of hypertensive patients [26,27,28,29,30].

VonAchen et al. compared, based on computed tomography angiography (CTA) and magnetic resonance imaging (MRI) scanning, the prevalence of accessory renal arteries in patients with resistant hypertension undergoing RDN with normotensive subjects undergoing screening as possible renal transplant donors. Accessory renal arteries were markedly over-represent in hypertensive patients than healthy controls (59% vs. 32%). A total of 29% of patients undergoing the RDN treatment do not respond to therapy. Patients without accessory arteries exhibited a statistically insignificant elevated rate of response to RDN compared to individuals possessing at least one additional vessel (83% vs. 62%). Furthermore, they demonstrated a significantly greater response to RDN when compared to patients with untreated accessory arteries (83% vs. 55%) [7]. Song et al. investigated the anatomy of renal vascularity in 314 consecutive patients undergoing living donor nephrectomy. Computed tomography angiograms revealed at least one accessory renal artery in 37% of kidney donors, and 22% of donors had early-branching renal arteries. Most of these vessels had a diameter of less than 3 mm. Such a high variability of renal artery anatomy may significantly impact the RDN procedure’s effectiveness because these vessels were too small to allow complete denervation in their range [36]. 

Okada et al. categorized the renal arteries based on the anatomical criteria of qualifying resistant hypertension patients for renal sympathetic denervation. One hundred twenty-two consecutive RTH patients were assessed by computed tomography angiography. Renal artery anatomy was divided into two types: type A with a main renal artery ≥20 mm long and ≥4.0 mm in diameter, or type B with a main renal artery <20 mm in length or main renal artery <4.0 mm in diameter. The A type contained three subtypes: A1 without accessory renal arteries, A2 with accessory renal arteries <3.0 mm in diameter, and A3 with accessory renal arteries ≥3.0 mm in diameter. It was revealed that 41% of patients had at least one accessory renal artery. The average diameter of the accessory renal arteries or early branches was 3 mm on the right side and 2.7 mm on the left side. Okada et al. likewise found a slightly greater prevalence of accessory renal arteries (41%) in RTH patients than reported in the general population (22–30%) [27].

In the initial clinical trials, accessory renal arteries and early branches were an exclusion criterion [4,26,27,28,29,30,31]. The disappointing reduction in BP observed raised doubts regarding the effectiveness of renal denervation in clinical practice. This uncertainty stemmed from the fact that sympathetic nerve fibers surrounding the renal artery extend beyond the depth of energy penetration achievable from the lumen of the main artery. Consequently, the previous catheter-based ablation method fell short of complete denervation, and increasing the energy for deeper penetration posed the risk of intima injury [36].

### 4.2. Accessory Renal Artery Periarterial Renal Sympathetic Nerves

In recent years, new studies have allowed us to understand the anatomy of the renal arteries’ sympathetic nerve fiber to a greater extent. They may improve the effectiveness of the denervation of renal arteries. Sakakura et al. showed that in the distal segments of the renal arteries, there are fewer periarterial nerve fibers, but they are located closer to the lumen of the vessel than in proximal segments. These results may be helpful in choosing the appropriate strategy for performing the renal denervation procedure to increase its effectiveness. Performing ablation in the proximal and middle fragments of the vessels allows for damaging more nerve fibers because they are more concentrated in this area. However, ablation in the distal segment requires less thermal energy because the nerve fibers are located there more shallowly.

Moreover, the authors assessed that 75% of nervous fibers were located in the range of 5 mm of the artery lumen, implying that reaching an ablation deepness of 5 mm results in damage to about 80% of nerve fibers [36,37,38].

Finn et al. was the first paper to focus on the characteristic of the nerve fibers running along the accessory renal arteries. Based on examining kidneys and surrounding tissues during autopsy as well as a histological evaluation conducted using morphometric software, Finn et al. found significant differences in the morphology of the nerve fibers in the adventitia of the renal arteries and accessory renal arteries. Nerves associated with the main and accessory arteries have similar sizes. However, there were more nerve fibers around the main renal arteries, but the fibers surrounding the accessory arteries were closer to their lumen. Moreover, Finn et al. stated that there are more differences between main and accessory renal arteries. The number of nerve fibers located in the adventitia of the main renal arteries decreases from the proximal to the distal segments; however, the number of nerve fibers associated with the accessory renal arteries remains mostly unchanged. These findings make the accessory renal artery an essential target for RDN and may encourage the development of new ablation methods. The relatively large number of nerve fibers in the distal segments of the accessory arteries and the fiber’s proximity in these segments prompts us to focus on developing tools that enable ablation in small-diameter vessels. Addressing accessory renal arteries in the next clinical studies may enhance the effectiveness of RDN [28,37].

Present knowledge on the relevance of anatomical parameters on future changes in blood pressure after RDN is still limited, although it is essential for ongoing and subsequent clinical studies. The absence of dedicated markers of treatment success to confirm that denervation has been completely achieved remains one of the significant challenges [5,6,26,27,28,39,40,41].

### 4.3. Percutaneous Renal Denervation among Patients with Anatomical Variations of Renal Arteries

The available literature shows many examples of sympathetic renal denervation among patients with accessory renal arteries. In most cases, the time to evaluate the procedure’s efficacy is short, so further, more extensive studies are required to define the long-term effects of the treatment. Imbalzano et al. demonstrated an interesting case of a fifty-two-year-old woman with resistant hypertension treated with seven antihypertensive drugs. Selective angiography of the renal arteries showed the presence of an additional right renal artery. The patient underwent bilateral renal artery denervation using The Symplicity catheter. Three months after the procedure during a follow-up visit, the patient’s blood pressure was normal, and the patient took only one antihypertensive drug. [42].

Atas et al. reported a 42-year-old female patient with long-term hypertension that was resisted pharmacological therapy with five different antihypertensive drugs. Angiography revealed a typical morphology of the right renal artery. While on the left side, there was a well-developed left accessory renal artery with a diameter above 4 mm and length above 20 mm. Therefore, three vessels were denervated using a radiofrequency ablation catheter. One month after the procedure, a follow-up visit was carried out. The patient’s blood pressure was found to be normal, and she additionally required the use of only one antihypertensive drug [43].

A more extended observation period is available in the case presented by Bertoldi et al. A 61-year-old man had many years of history of uncontrolled hypertension despite using six antihypertensive drugs. The patient was referred for magnetic resonance angiography (MRA), which demonstrated two left accessory arteries arising straight from the abdominal aorta. Percutaneous renal sympathetic denervation was performed only in the main renal arteries due to the small diameter of the accessory renal arteries.

At the follow-up visit, twelve months after the procedure, ABPM showed a significant reduction in blood pressure. In addition, adequate blood pressure control required using only three antihypertensive drugs, while he was using six prior to the procedure [44].

De Leon-Martinez et al. presented the case of a 55-year-old man with a 13 year history of poorly controlled hypertension. He used four antihypertensive drugs at therapeutic doses. An aortography showed a proximal bifurcation in the left renal artery close to the ostium, a small right renal artery that supplies only the cranial pole, and a dominant right accessory artery that originates close to the aortic bifurcation. Afterward, computed tomography (CT) was performed. CT scans confirmed that the dominant right artery took off from the common iliac artery and that the left renal artery has the proximal bifurcation near the ostium. The patient was qualified for catheter-based percutaneous renal sympathetic denervation. He had eight ablations in each of the renal and accessory arteries. ABPM performed two months after the RDN procedure demonstrated a significant decrease in blood pressure: 50 mmHg in systolic and 20 mmHg in diastolic BP [45].

As postulated by many researchers, the presence of accessory renal arteries may also be the cause of secondary hypertension. Chan and Florence Hui Sieng Tan presented two cases of patients with secondary hypertension. They suggest that renin-dependent hypertension was caused by accessory arteries. Both patients with a long-term history of hypertension were admitted to the hospital due to high blood pressure and hypokalemia. During diagnostics, both patients had an elevated renin level with a normal aldosterone-to-renin ratio (ARR). Renal magnetic resonance angiography (MRA) showed additional renal arteries in both patients. The patients were diagnosed with secondary hypertension. An aldosterone antagonist was added to the treatment, normalizing blood pressure. Treatment with aldosterone antagonists has been successful in these patients. However, despite drug treatment, blood pressure remains high in some patients. The authors postulate that renal artery denervation may be effective in patients with resistant secondary renin-dependent hypertension, especially those with accessory renal arteries [46].

**Figure 4:**
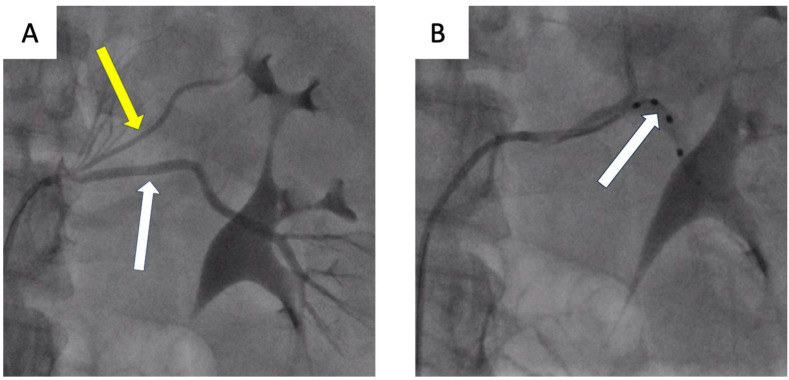
Patient 1. (**A**) Left renal artery angiography. Patient with resistant hypertension and multiple renal arteries. Angiography showing the left main renal artery (white arrow) and left accessory artery (yellow arrow). (**B**) Radiofrequency ablation was performed only in the left main renal artery due to the small diameter of the accessory artery. The Symplicity Spyral (Medtronic, Minneapolis, MN, USA) multi-electrode renal denervation catheter (white arrow).

**Figure 5:**
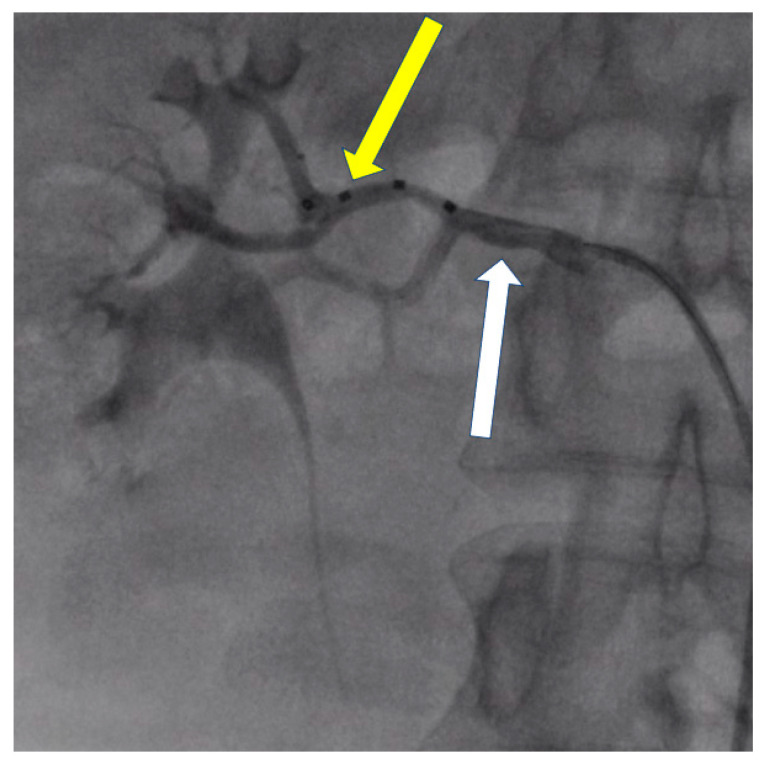
Patient 2. Right renal artery angiography. Angiography showing the main renal artery (white arrow) with two branches. Radiofrequency ablation was performed in both branches with the Symplicity Spyral (Medtronic, Minneapolis, MN, USA) multi-electrode renal denervation catheter (yellow arrow).

**Figure 6:**
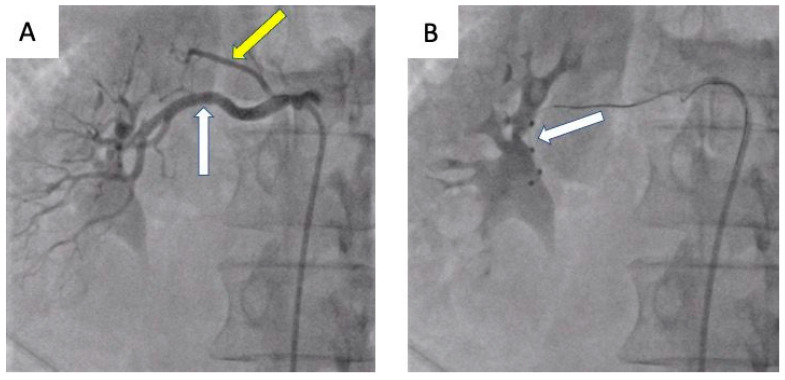
Patient 3. (**A**) Right renal artery angiography showing the right main renal artery (white arrow) and early branch of the renal artery (yellow arrow). (**B**) Right renal artery angiography showing right renal artery denervation. The Symplicity Spyral (Medtronic, Minneapolis, MN, USA) multi-electrode catheter (white arrow). Radiofrequency ablation was performed in the right main renal artery. The accessory renal artery was not ablated due to its small diameter.

The presented cases demonstrate patients who, due to vascular anatomy or other factors, were not optimal candidates for denervation and, using the initial criteria, were not eligible for clinical trials. However, the procedures performed resulted in a significant pressure decrease in the short-term control (Table 1). It is postulated that further research and longer follow-up are needed to increase the number of patients in whom renal denervation may be successful. To enhance the presentation of the percutaneous renal denervation procedure, this article presents intraoperative images obtained during procedures conducted at the University Hospital in Krakow (Figure 4, Figure 5 and Figure 6).

### 4.4. The Effectiveness of Renal Denervation in Patients with Anatomical Variations of Renal Arteries

Several studies evaluated the effectiveness of renal artery denervation in patients with accessory renal arteries. Id et al. conducted a survey in which renal denervation was performed on 74 patients. Patients were split into two groups: Group I (54 patients) with bilateral single renal arteries and Group II (20 patients) with accessory renal arteries. In addition, patients with ARA were divided into two other groups. In Group IIa, all renal arteries were denervated. On the contrary, in Group IIb, none or only some of the accessory renal arteries were denervated. After 6 months, blood pressure reduction was significantly more effective in patients with bilateral single renal arteries (Group I) [9]. In Group I, six months after the intervention, office BP reduction was 17/7 mm Hg, whereas, in Group II, the office systolic and diastolic BP reductions were 6/0 mmHg. Group IIa had an office BP reduction of 9/1 mm Hg and Group IIb of 4/1 mm Hg. The study showed a significant difference in the systolic and diastolic office BP decrease between Group I and Group II patients. Significant differences were observed in systolic office BP changes between patients with bilateral single renal arteries and patients with accessory renal arteries and incomplete or no denervation of the accessory arteries (Group IIb). However, there was no significant difference in the systolic BP decrease between patients with bilateral single renal arteries (Group I) and patients with accessory renal arteries and total denervation of all arteries (Group IIa) [9].

Bartus et al. analyzed the long-term result of renal denervation in patients with accessory renal arteries (7 patients) and bilateral single renal arteries (24 patients). Before the procedure and at 6, 12, 24, and 36 months after, their BP was measured. In Group 1, a notable reduction in systolic BP was observed, whereas no variations in diastolic BP were found. Group 2 had significant differences in systolic and diastolic BP reductions. There were no significant differences in mean systolic and diastolic BP between the study groups 36 months after renal denervation. However, as in the study by Id et al., blood pressure reduction was more effective in patients with bilateral single renal arteries in this long-term observation blood. As mentioned, renal sympathetic nerves are located in the adventitia of all renal arteries, both accessory renal arteries and bilateral single renal arteries. Therefore, incomplete damage of the renal sympathetic fibers in patients with accessory renal arteries seems to be the possible explanation for differences in the effectiveness of the treatment between the groups. A more conservative approach is taken in smaller arteries due to safety concerns regarding circumferential catheter manipulation, or it is attributed to the inability to denervate all accessory renal arteries [25].

Frequently, accessory renal arteries, including dual renal arteries and early polar branches of renal arteries, have a size smaller than 4 mm. As a result, these arteries were frequently not initially accessible for ablation using approved devices. The incomplete denervation may lead to a less effective reduction in blood pressure than in cases of a single renal artery [9,25].

Contrary to the previous results, Ewen et al. did not find a relation between the change in systolic blood pressure (SBP) and the presence of ARA. Ewen et al. assessed renal artery anatomy among 564 patients qualified for renal artery denervation. An accessory renal artery was present in 30% of patients. After six months, blood pressure was significantly reduced by 19/8 mmHg regardless of the presence of accessory renal arteries. Patients with a bilateral diameter of renal arteries ≤4 mm had a more prominent decrease of SBP compared to patients with a unilateral diameter ≤4 mm or a bilateral diameter >4 mm (−29 vs. −26 vs. −17 mmHg). After six months, neither the length of the renal artery nor the number of RF ablations impacted blood pressure reduction [47].

### 4.5. Contemporary Trials

As new catheters were invented and the following data were collected, patients with multiple renal arteries were included in clinical trials. Several new renal artery denervation catheters have been introduced in recent years that will allow for more complete ablation. All randomized clinical trials conducted in recent years using new-generation catheters have confirmed the effectiveness of renal artery denervation in lowering blood pressure [5,6,21,39].

The SPYRAL HTN-OFF MED and ON-MED trials were performed using radiofrequency ablation with multipolar Symplicity Spyral catheters (Medtronic, Minneapolis, MN, USA). This catheter type administers up to four ablations simultaneously in a spiral pattern by creating heat using high-frequency electric energy. All renal arterial branch vessels, including accessory, branch, and main renal arteries located outside the renal parenchyma, with diameters ranging from 3 to 8 mm, underwent ablations [17,18,48].

The RADIANCE-HTN SOLO and RADIANCE-HTN TRIO trials were performed using the Paradise catheter (ReCor Medical, Palo Alto, CA, USA). This balloon-cooled device makes a fully circumferential thermal ablation pattern using acoustic energy. Multiple ablations run of 1 min were provided to the main renal artery from distal to proximal. Ultrasound energy was used for complete circumferential thermal ablation of the renal sympathetic nerve, targeting a depth of 6–7 mm, corresponding to the anticipated location of sympathetic nerves within the adventitia of the primary renal artery. As a result, the need for denervation of branch renal arteries was effectively eliminated.

The RADIANCE-HTN SOLO trial excluded patients with a main renal artery diameter of less than 4 mm or greater than 8 mm, a main renal artery length of less than 25 mm, and accessory renal arteries with a diameter of less than 2 mm or greater than 8 mm.

Patients with a main renal artery diameter of less than 3 mm or greater than 8 mm, a main treatable renal artery length of less than 20 mm, which may include proximal branching, and accessory renal arteries with a diameter of less than 2 mm or greater than 8 mm were excluded from the RADIANCE-HTN TRIO trial [19,20,49].

The TARGET BP OFF-MED and TARGET BP I trials were performed using the Peregrine Catheter and dehydrated alcohol, United States Pharmacopeia (USP), as the pharmaceutical element. The Peregrine Catheter has been created to accommodate the standard range of renal artery diameters from 4 to 7 mm. Its goal is to administer alcohol to the perivascular region of the renal arteries, enabling the ablation of sympathetic nerves located in adventitia [22].

## 5. Conclusions

In recent years, there has been an increased interest in percutaneous renal denervation in hypertensive patients. The development of new-generation catheters and the advancement in knowledge of renal arteries anatomy may improve the effectiveness of treatment and reduce the number of patients who do not respond to treatment. Comprehending the influence of accessory renal arteries on hypertension, especially resistant hypertension, still needs to be completed. Imaging tests such as computed tomography and magnetic resonance imaging allow for better anatomical understanding of these vessels and optimization of the patient’s qualification for invasive treatment of hypertension. However, further clinical trials and the development of treatment methods for small-diameter vessels are needed to increase the number of patients who will benefit from the ablation of renal sympathetic nerves.

## Figures and Tables

**Table 1:** Effects of renal denervation on blood pressures. A summary of presented case reports.

Author	Patient	Variations of Renal Arteries	Intervention	Reduction in Hypertension Therapy	Reduction in Blood Pressure [mmHg]
Imbalzano, E et al. [42]	52-yo woman	Right ARA	RDN, RF ablation catheter (Symplicity, Medtronic)	Seven to one drug	no data available
Atas, Halil et al. [43]	42-yo woman	Left ARA	RDN, RF ablation catheter (Symplicity, Ardian, Medtronic)	Five to two drugs	−55/20 after one month
Bertoldi, Letizia et al. [44]	61-yo man	Two small left ARA’s	RDN, RF ablation catheter (Symplicity, Ardian Medtronic)	Six to three drugs	−16/10 after one month, −45/24 after 12 months
de Leon-Martinez, Enrique Ponce et al. [45]	55-yo man	Proximal bifurcation in the left RA and right ARA	RDN, RF ablation catheter (Symplicity Medtronic)	Five to two drugs	−29/9 after one month, −50/20 after two moths
Chan, Pei Lin, and Florence Hui Sieng Tan. [46]	21-yo woman	Bilateral ARA’s	Spironolactone	no data available	no data available
	41-yo woman	Left ARA			
